# The effect of impact location, velocity, and frequency on head injury criterion during ice hockey helmet drop tests

**DOI:** 10.7717/peerj.21366

**Published:** 2026-06-08

**Authors:** Rui Lin, Haiyang Hu, Tianpei Du, Xinglong Zhou, Ronghui Wang, Xianglin Wan

**Affiliations:** 1 School of Sport Science, Beijing Sport University, Beijing, China; 2 Foshan Supervision Testing Centre of Quality and Metrology, Foshan, China

**Keywords:** Impact location, Ice hockey helmet, Head injury criterion, Abbreviated injury scale, Concussion

## Abstract

**Background:**

This study investigated whether differences in ice hockey helmet protection performance at varying impact velocities influence head injury risk.

**Methods:**

Linear acceleration data were recorded during drop tests on 24 ice hockey helmets by using a uniaxial accelerometer in a collision test machine under low- and ambient-temperature environments for sequential low-, medium-, and high-velocity impacts. Head injury criterion (HIC), seasonal injury risk (SIR) under different impact velocities, and total seasonal injury risk (TSIR) were calculated for each helmet location.

**Results:**

In both temperature environments, the back, front, and side locations produced higher TSIR than the top. Under low-velocity impacts, the side location produced higher SIR and HIC than the back and front. Under medium- and high-velocity impacts, the side location produced lower SIR than the back and front, or showed no difference compared to the back. Except for comparisons between the side and back/front under low-velocity impacts, SIR and HIC differences across locations were inconsistent or nonsignificant.

**Conclusion:**

These findings suggest that under low-velocity impacts, the higher HIC at the side location may be a key factor influencing head injury risk. To enhance helmet protective performance, optimizing energy absorption at the side location during low-velocity impacts is recommended.

## Introduction

Ice hockey is a high-risk winter sport with a reported head injury incidence ranging from 0.90 to 2.60 per 1,000 athlete exposures (Tuominen et al., 2015; Tuominen et al., 2016). Head injuries are predominantly classified as Abbreviated Injury Scale (AIS) score of 2 and are often diagnosed as concussion, typically presenting with headache and loss of consciousness (Cassidy et al., 2004; Owattanapanich et al., 2023). In more severe cases, symptoms may persist for several years (Tator, Blanchet & Ma, 2023). Although ice hockey helmets are designed as wearable protective equipment to reduce the risk of head injuries and have proven effective in preventing superficial trauma, their ability in preventing brain injuries remains limited (Schneider et al., 2017). Therefore, improving helmet performance to reduce the risk of AIS 2 head injuries is crucial (McIntosh, 2012).

In ice hockey, the incidence of athlete head injuries may be influenced by multiple factors including the impact location of the helmet, impact velocity, impact frequency, and helmet protective performance (Rowson, Rowson & Duma, 2015). Delaney, Al-Kashmiri & Correa (2014) reported that concussion incidences following impacts to the side, back, top, and front locations of a helmet were 37.3%, 17.7%, 3.9%, and 2.0%, respectively. Comparative studies of head responses across different impact locations have shown that lateral impacts to an unhelmeted head tend to produce greater head acceleration or brain tissue strain (Fanton, Sganga & Camarillo, 2019; Walsh, Rousseau & Hoshizaki, 2011). In addition, the relatively weaker protection on the lateral side of helmets may result in increased head acceleration during impact (Pearsall, Lapaine & Ouckama, 2016; Post et al., 2013). These findings may help explain the higher incidence of concussions associated with side impacts and may provide insights for optimizing helmet design to reduce the risk of head injuries. However, these studies employed head impacts at a single velocity and did not evaluate helmet protective performance across a range of impact velocities, such as low (2–4 m/s), medium (4–6 m/s), and high (6–8 m/s) impact velocities reported in real-game situations (Meliambro et al., 2022; Post et al., 2019). If the density and material of the helmet liner are not appropriately matched to the impact velocity, the foam may undergo either insufficient or excessive deformation, thereby transmitting the impact forces directly to the head (Mills et al., 2021; Valevicius et al., 2024). As the impact velocity changes, the energy absorption performance of helmet cushioning designs varies accordingly, consequently influencing the risk of head injury for athletes when different head locations are impacted. Although Levy et al. (2021) considered multiple impact velocities, the study did not provide statistically supported evidence of the differences in protective performance across helmet locations. In addition, these studies did not consider the effect of impact frequency when evaluating helmet-protective performance. In ice hockey, many head impacts occur as secondary events following an initial body collision. For example, a player may be unexpectedly subjected to a lateral body check due to blind spots in the visual field (Aguiar et al., 2020). Without adequate bracing or reflexive postural responses, this unexpected contact may cause the lateral aspect of the head to subsequently strike the glass (Aguiar et al., 2020). These impact characteristics may contribute to the higher frequency of impacts to the sides of the head compared with those to the top, back, or front (Aguiar et al., 2023; Aguiar et al., 2020). Variations in impact frequency across helmet locations may influence the risk of head injuries, and the effectiveness of helmet design optimization could be reduced if the weighting assigned to impact frequencies at different locations is not appropriately considered (Fanton, Sganga & Camarillo, 2019). Therefore, a comprehensive evaluation of ice hockey helmet performance should incorporate both impact velocity and frequency across locations.

During linear acceleration, an impact to the head induces relative displacement between the skull and brain tissue owing to differences in inertia, generating a pressure gradient characterized by compression and positive pressure at the impact site, and tension and negative pressure on the opposite side (Namjoshi et al., 2013). When intracranial pressure exceeds the tolerance threshold, individuals may develop clinical symptoms associated with concussion, such as headache, dizziness, and nausea (Haider et al., 2018). The Head Injury Criterion (HIC) was initially developed to evaluate the risk of skull fractures (Davies & Gugliotta, 2012). Subsequent cadaveric experiments further established its functional relationship with the AIS, which allows HIC to predict the probability of AIS 2+ head injuries under a single impact based on the linear acceleration injury mechanism described above (Davies & Gugliotta, 2012). Incorporating both the velocity and frequency characteristics of head impacts in ice hockey, the HIC enables the evaluation of the seasonal injury risk (SIR) at different impact velocities for each helmet location, as well as the calculation of the cumulative SIR across low-, medium-, and high-velocity impacts, defined as the total seasonal injury risk (TSIR) (Rowson, Rowson & Duma, 2015). By comparing HIC values across different helmet locations and examining the consistency among HIC, SIR, and TSIR values, it is possible to identify variations in protective performance at different helmet locations under different impact velocities, and assess the effect of impact velocity and frequency on injury risk. However, it remains unclear which specific helmet locations provide insufficient protective performance at certain impact velocities, thereby increasing the risk of head injuries. Ice hockey helmets are used not only in standard ice rinks with relatively low temperatures but also during off-ice skating simulations or training activities conducted on roller or dryland surfaces under ambient-temperature conditions (Wagner et al., 2021). This study investigated the effects of impact location, velocity, and frequency on HIC during ice hockey helmet drop tests following low- or ambient-temperature treatment, with the goal of determining whether differences in helmet protection performance at low-, medium-, and high-velocity impacts are key factors influencing the risk of head injuries. These findings will support helmet manufacturers in optimizing their designs for improved impact protection. This study hypothesized that TSIR and SIR would decrease in the order of side > front > back > top, and that HIC would decrease in the order of side > top > front > back across all impact velocities and temperature conditions.

## Materials & Methods

### Ice hockey helmet

A total of 24 ice hockey helmets were purchased for drop testing, including the RE-AKT (200, 150, 100, 95, 85, and 75), RE-AKT, HYPERLITE, IMS (7.0 and 5.0), and 5100 models from BAUER; the TACKS (710, 310, 210, and 110), FL (90, 60, and 40), FITLITE 3DS, and RESISTANCE 300 models from CCM; the COVERT (PX+ and RS PRO) models from WARRIOR; the X73 model from IBX; and the only ice hockey helmet from BAUD. The cushioning liners of helmets differ in material composition, mechanical structure, foam density, and thickness. Large-sized helmets were selected for standardization; however, owing to stock limitations, models RE-AKT (200 and 95), HYPERLITE, RE-AKT, RESISTANCE 300, and FL 40 were purchased in small sizes, and the RE-AKT 150 and IMS 7.0 models were purchased in medium sizes.

### Data collection and reduction

Equipping the headform with both a helmet and a facemask better represents real-world playing conditions, and previous studies have shown that the addition of a facemask can increase HIC values (Rush et al., 2017). However, if the facemasks were retained, some helmets might contact the facemask before the helmet shell during frontal impacts, which could introduce greater confounding effects compared to tests conducted without the facemask when comparing different impact locations. Therefore, the facemasks were removed to ensure consistent impact contact conditions across helmet models (Bartsch et al., 2012). The helmets were subsequently placed in an HH-480D test chamber (Dongguan Huahong Instrument, Guangdong, China) maintained at −25 ± 2 °C for 4 h to simulate the low-temperature environments experienced during real-world ice hockey gameplay. After low-temperature treatment, the helmets were immediately subjected to drop testing using a 100A-1600KG twin-wire impact test machine (Cadex Inc., Saint-Jean-sur-Richelieu, Quebec, Canada). A uniaxial accelerometer (Model 353B18, PCB Piezotronics Inc., Depew, NY, USA) mounted at the center of mass of the headform was used to record the linear acceleration during impact.

**Figure 1:**
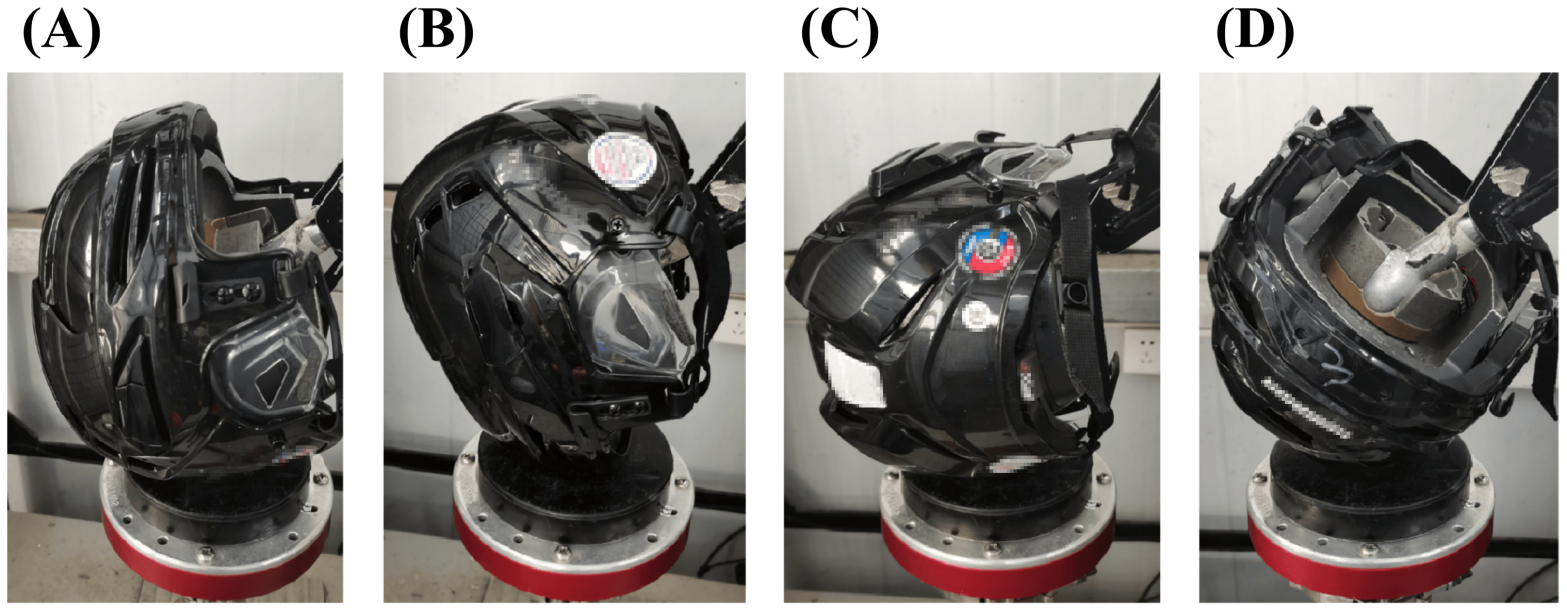
Impact locations of ice hockey helmet. (A) back, (B) front, (C) side, and (D) top views.

K1A magnesium–aluminum alloy half-headforms conforming to European Committee for Standardization EN 960 (2006) standard were selected based on the helmet size, and the helmets were fitted onto the headforms. During fitting, the protective area of each helmet was ensured to fully cover the surface of the headform, and the helmet edge was aligned with the edge of the headform to avoid improper fitting such as forward fit or backward fit. The helmet retention system was then adjusted to minimize relative motion between the helmet and the headform and to prevent detachment during impacts. Helmets and headforms were mounted and adjusted to align with four common impact locations (Fig. 1) (Rowson, Rowson & Duma, 2015).

In accordance with the International Organization for Standardization ISO 10256-2, 2024 standard and the experimental protocol developed by Rowson, Rowson & Duma (2015), impacts were conducted in descending order of frequency, with the helmet, headform, and associated components impacting a modular elastomer programmer (MEP) at velocities of 3.0 (low), 4.6 (medium), and 6.1 m/s (high), respectively. A helmeted headform was dropped onto a MEP to simulate typical head impact scenarios encountered in ice hockey. Each location of the helmet was impacted once at each velocity with 30–90 s intervals between consecutive impacts at the same location (Ouckama & Pearsall, 2012). The impact velocity was controlled by adjusting the initial drop height of the helmet and monitored using a laser velocimeter, with an allowable measurement error of ±0.09 m/s. The MEP had a thickness of 2.54 cm, a diameter of 15.24 cm, and a hardness of 59.5 Shore A. Both the velocity control and MEP specifications complied with the International Organization for Standardization ISO 10256-2 (2024) standard. Additionally, to maintain the helmet at a low temperature, it was subjected to cold treatment for more than 20 min after every three impacts at each location in accordance with the National Highway Traffic Safety Administration FMVSS 218 (2011) standard.

The ambient-temperature impact test was conducted 24 h after completing the low-temperature impact test. The helmets were placed in a chamber maintained at 20 ± 3 °C for 4 h to simulate the ambient-temperature environments experienced during off-ice skating simulations or training activities. The procedure was identical to that used in the low-temperature test, except for the environmental temperature. The low- and ambient-temperature treatment methods complied with the International Organization for Standardization ISO 10256-1 (2024) standard. All procedures were performed by experienced quality control personnel.

The HIC is a metric for assessing the risk of AIS 2+ head injuries in ice hockey based on the linear acceleration injury mechanism (Davies & Gugliotta, 2012). Therefore, the recorded linear acceleration data were filtered using a CFC 1000 filter in accordance with The SAE International SAEJ 211-1 (2022). The HIC was calculated for each impact, and the SIR and TSIR values for each impact location were subsequently calculated using the weighting coefficients of different impact conditions (Davies & Gugliotta, 2012; Rowson, Rowson & Duma, 2015), as defined by the following equations:

(1) \begin{eqnarray*}\mathrm{HIC}= \left\{ { \left[ \frac{1}{ \left( {t}_{2}-{t}_{1} \right) } \int \nolimits \nolimits _{{t}_{1}}^{{t}_{2}}a \left( t \right) \mathrm{d}t \right] }^{2.5} \left( {t}_{2}-{t}_{1} \right) \right\} _{\mathrm{max}}\end{eqnarray*}

(2) \begin{eqnarray*}R \left( \mathrm{HIC} \right) = \frac{1}{1+{\mathrm{e}}^{ \left( 2.49+ \frac{200}{\mathrm{HIC}} -0.00483\mathrm{HIC} \right) }} \end{eqnarray*}

(3) \begin{eqnarray*}\mathrm{SIR}=E \left( \mathrm{L},\mathrm{S} \right) R \left( \mathrm{HIC} \right) \end{eqnarray*}

(4) \begin{eqnarray*}\mathrm{TSIR}=\sum _{\mathrm{S}=1}^{3}E \left( \mathrm{L},\mathrm{S} \right) R \left( \mathrm{HIC} \right) \end{eqnarray*}

where HIC is represented in g^2.5^s, *a* represents the linear acceleration (g), and *t* represents time (s). The values *t*_1_ and *t*_2_ indicate the start and end times that produce the maximum HIC, respectively. *R* (HIC) is the probability of sustaining an AIS 2+ head injury from a single-head impact (Davies & Gugliotta, 2012). *E* (L, S) is a weighting coefficient for each impact condition (the expected number of impacts per season), where L is the impact location and S is the impact velocity type (Table 1). SIR and TSIR are the injury probabilities calculated from the HIC. For the same metric, the same equation is used for both low- and ambient-temperature environments. The impact locations, velocities, and frequency weighting coefficients used in this study were derived from head impact data collected from male and female collegiate and high school ice hockey players (Rowson, Rowson & Duma, 2015). The calculated HIC, SIR, and TSIR values can be used to evaluate the protective performance of helmets used in both adult and youth hockey.

**Table 1:** Weighting coefficients of different impact conditions.

Impact location	Impact velocity type		
	Low	Medium	High
Back	61.4	4.5	2.2
Front	62.9	4.6	0.6
Side	65.6	2.2	0.3
Top	21.5	1.1	0.1

### Statistical analysis

Normality and homogeneity of variance were assessed using the Shapiro–Wilk and Levene’s tests. If assumptions were satisfied, one-way ANOVA with Bonferroni *post hoc* tests was employed to compare TSIR, SIR, and HIC values across different helmet locations under different impact conditions. If assumptions were violated, the Friedman test was employed, and Bonferroni-adjusted *p*-values were used for *post-hoc* comparisons. To determine whether differences in helmet protection performance at low, medium, and high impact velocities were the key factors influencing the risk of head injuries, this study evaluated the consistency among the three metrics and compared the HIC across different helmet locations. Statistical significance was defined as the type I error rate ≤ 0.05, and all statistical analyses were performed using SPSS 26.0 (IBM Corp., Armonk, NY, USA).

## Results

### Effects on TSIR values

The impact location had a significant effect on the TSIR values in both low- (*χ*^2^ = 47.000, *p* < 0.001) and ambient-temperature environments (*χ*^2^ = 44.450, *p* < 0.001) (Table 2). *Post hoc* analyses revealed that the back, front, and side locations produced higher TSIR values than the top location in both temperature environments (*p* < 0.001).

**Table 2:** The TSIR values calculated for different impact locations on the helmets.

Temperature environment	Impact location				*p*
	Back	Front	Side	Top	
Low	6.6(5.2,8.8)^c^	5.7(4.9,7.9)^c^	7.8(7.0,9.8)^c^	2.5(1.7,3.0)	<0.001
Ambient	6.2(4.9,7.1)^c^	5.7(4.3,6.4)^c^	5.2(4.1,6.9)^c^	1.9(1.5,2.5)	<0.001

**Notes.**

c indicates a significant difference compared with the top impact location.

### Effects on HIC and SIR values

**Figure 2:**
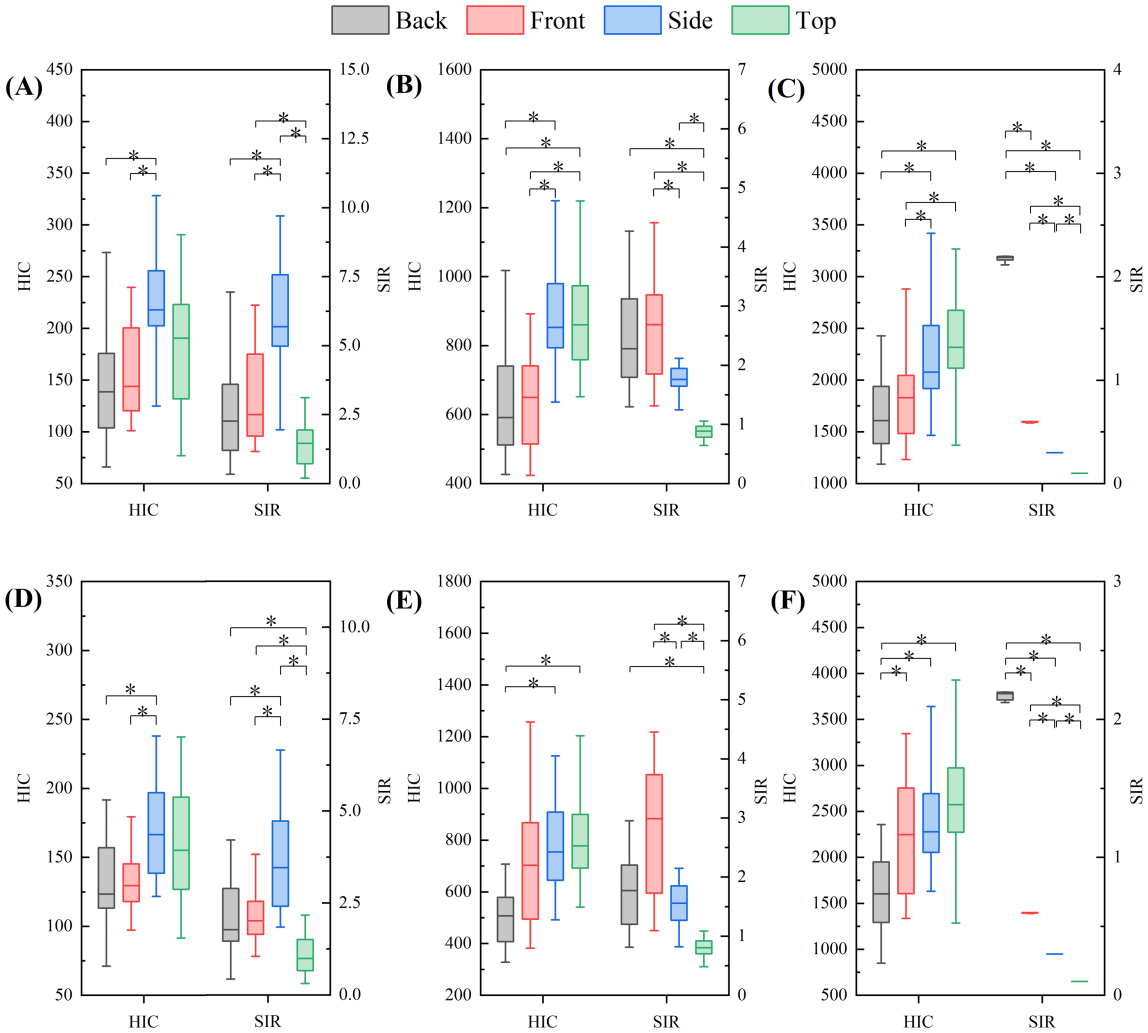
HIC and SIR values of different helmet locations under different conditions. Low temperature impacts at (A) low-velocity, (B) medium-velocity, and (C) high-velocity. Ambient temperature impacts at (D) low-velocity, (E) medium-velocity, and (F) high-velocity. * Indicates statistical significance (*p* < 0.05) to a 95% confidence level.

**Figure 3:**
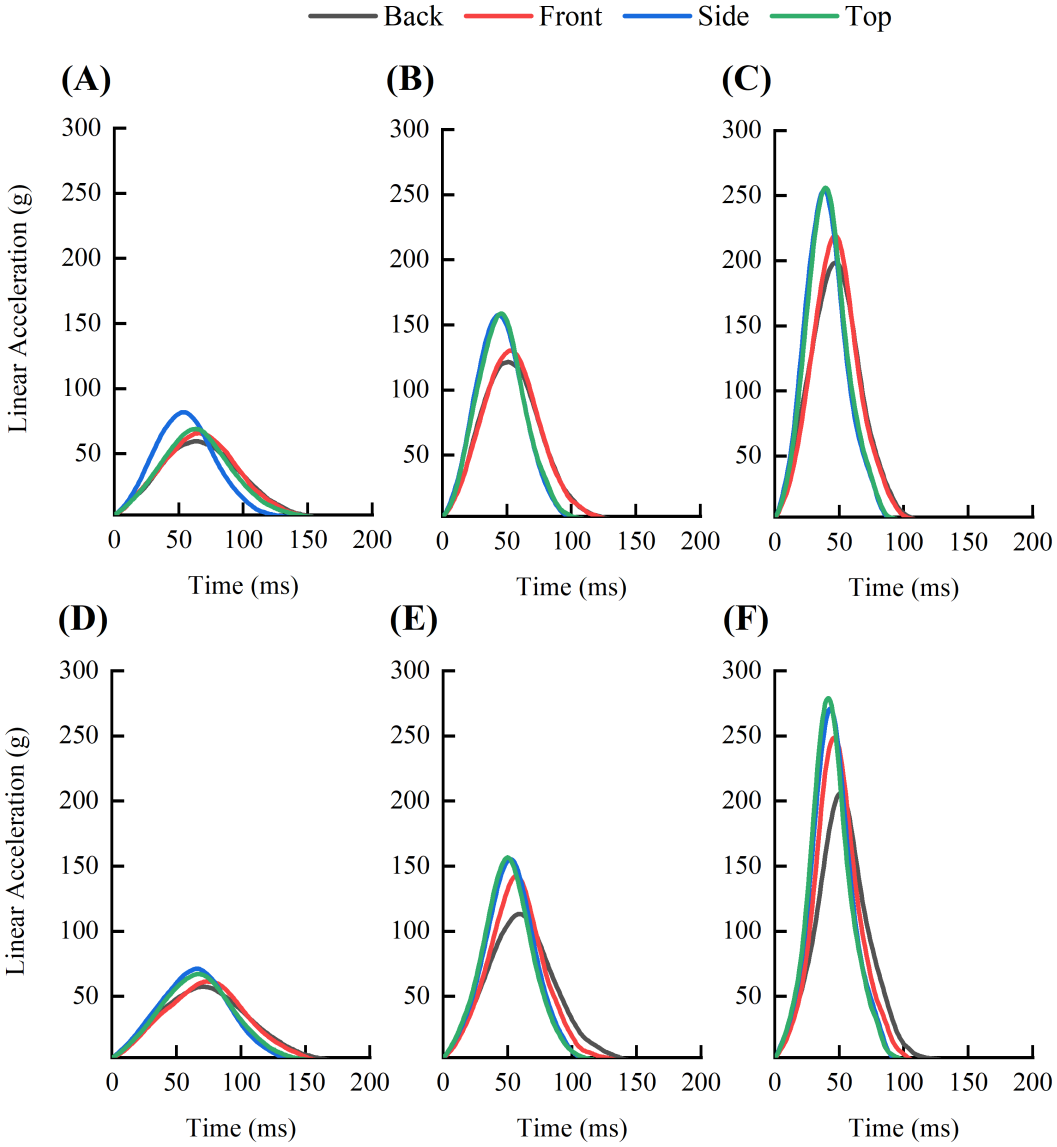
Linear acceleration curves of head impacts at different helmet locations under different conditions. Low-temperature impacts at (A) low-velocity, (B) medium-velocity, and (C) high-velocity. Ambient temperature impacts at (D) low-velocity, (E) medium-velocity, and (F) high-velocity.

In the low-temperature environment, the impact location had a significant effect on the HIC at low (*χ*^2^ = 22.750, *p* < 0.001), medium (*χ*^2^ = 34.550, *p* < 0.001), and high (*χ*^2^ = 45.800, *p* < 0.001) velocities. Similarly, the SIR values were significantly affected at low (*χ*^2^ = 39.650, *p* < 0.001), medium (*χ*^2^ = 52.650, *p* < 0.001), and high (*χ*^2^ = 72.000, *p* < 0.001) velocities. In an ambient-temperature environment, the impact location had a significant effect on the HIC at low (*χ*^2^ = 16.100, *p* = 0.001), medium (*χ*^2^ = 27.750, *p* < 0.001), and high (*χ*^2^ = 34.750, *p* < 0.001) velocities. Similarly, the SIR values were significantly affected at low (*χ*^2^ = 39.350, *p* < 0.001), medium (*χ*^2^ = 53.150, *p* < 0.001), and high (*χ*^2^ = 72.000, *p* < 0.001) velocities. *Post hoc* test results for both the HIC and SIR values are presented in Fig. 2. The linear acceleration curves of the helmet impacts are presented in Fig. 3.

## Discussion

The test parameters used in the present model were derived from real-world head-impact data collected during ice hockey competitions, which ensured that the TSIR and SIR values provided a more realistic assessment of helmet protective performance against the seasonal risk of AIS 2+ head injuries (Davies & Gugliotta, 2012; Rowson, Rowson & Duma, 2015). This study found that in low- and ambient-temperature environments, the back, front, and side locations produced higher TSIR values than the top location, whereas no significant differences were observed among the other locations. These results partially supported the research hypothesis, indicating that seasonal exposure to impacts on the back, front, and side locations of the helmet may increase the risk of AIS 2+ head injuries relative to the top location, whereas the injury risks across other locations may not differ significantly.

According to Eq. (4), the variation in TSIR value can be directly determined by the individual SIR values at each impact velocity (Rowson, Rowson & Duma, 2015). The back location produced a higher SIR value than the front location under a high-velocity impact, whereas no significant difference was observed under low- or medium-velocity impacts. This indicated that the SIR value variations under high-velocity impacts at these locations were likely minimal and may not have affected the TSIR value. The side location produced higher SIR values than the back and front locations under low-velocity impacts, whereas the opposite or no significant differences were observed under medium- and high-velocity impacts. This indicates that the TSIR value at the side location showed no difference compared to the back and front locations, which may be attributed to the net balancing of the SIR value variations across varying impact velocities. The back, front, and side locations produced higher SIR values than the top location, or the back location showed no difference from the top location. This indicates that the higher TSIR values observed at the back, front, and side locations compared to the top may be attributed to the cumulative effect of increased SIR values across different impact velocities.

According to Eq. (3), the SIR value at each impact velocity was calculated as the product of the impact frequency and risk of AIS 2+ head injuries, and its magnitude was determined by the impact frequency coefficient and HIC (Davies & Gugliotta, 2012; Rowson, Rowson & Duma, 2015). Under low-velocity impacts at low and ambient temperatures, the side produced higher HIC and SIR values than the back and front locations. The consistency of these results indicates that locational differences in helmet protection performance under low-velocity impacts may be a key factor influencing the seasonal risk of AIS 2+ head injuries. The increased SIR values observed at the side location relative to those at the back and front locations may have resulted in a higher TSIR value. This indicates that optimizing the energy absorption at the side of the helmet during low-velocity impacts may enhance the ice hockey helmet performance for protecting against AIS 2+ head injuries in ice hockey. Under low- and medium-velocity impacts at low and ambient temperatures, the HIC and SIR values showed no significant differences between the back and front locations. Similarly, no significant differences were observed between the back and top locations under low-velocity impacts at low temperatures. These consistent non-significant results indicate that, under these specific conditions, locational helmet protection and impact frequency may not affect the seasonal risk of AIS 2+ head injuries. Except for the comparisons discussed above, the HIC and SIR values across the helmet locations were inconsistent. This indicates that in most cases, differences in the frequency of impact across helmet locations may be a key factor influencing the seasonal risk of AIS 2+ head injuries.

Under all the impact velocity conditions, the top location exhibited the lowest impact frequency. When the back, front, and side locations produced higher SIR values than the top location, the corresponding HIC results were inconsistent with the SIR values. The higher TSIR values at these locations were attributed to the cumulative effect of the increased SIR values across different impact velocities. It is reasonable to infer that the relatively low impact frequency at the top location is a key factor for its lower TSIR value compared with other locations. Additionally, differences in impact frequency may be influenced by factors such as athlete sex, player position, and game type (Eckner et al., 2018), potentially explaining the inconsistencies observed between TSIR values and injury rates reported by Delaney, Al-Kashmiri & Correa (2014).

This study found that the side location produced a higher HIC than the back and front locations under low-velocity impacts at low and ambient temperatures, indicating that a single low-velocity impact on the side of a helmet may result in a higher risk of AIS 2+ head injury than an impact on the back or front. Under medium- and high-velocity impacts, the HIC varied between the low- and ambient-temperature environments. In low-temperature environments, the side and top locations produced a higher HIC than the back and front locations, indicating that a single medium- or high-velocity impact on the side or top of a helmet may result in a higher risk of AIS 2+ head injury than an impact on the back or front. In ambient-temperature environments, the side and top locations produced higher HIC than the back location, indicating that a single medium- or high-velocity impact on the side or top of the helmet may result in a higher risk of AIS 2+ head injury than an impact on the back. Additionally, the front produced a higher HIC than the back, indicating that a single high-velocity impact on the front of the helmet may result in a higher risk of AIS 2+ head injury than an impact on the back under ambient temperature conditions. In summary, the results of the HIC revealed the following ranking among helmet locations: side = top ≥ front ≥ back. These results partially support the research hypothesis, indicating that the side and top of the helmet may provide less effective protection against single impacts than the front and back locations.

The observed locational differences in the HIC may be attributed to helmet design and the anatomical structures of the skull and brain. During impact, the helmet shell dissipates approximately 10%–34% of the energy, and beyond preventing skull penetration, it facilitates the transmission and dispersion of forces by enlarging the effective energy-absorbing area of the liner, thereby reducing localized stress peaks (Fernandes & De Sousa, 2013). No visible structural damage, such as dents or cracks, was observed on the helmets during testing, indicating that the helmet shells used in this study possessed sufficient structural strength to withstand impacts and preserve the energy absorption capability of the inner liner. During the injection molding process, although the base material is the same throughout each helmet shell, variations in molding conditions, such as temperature, mold flow, and injection pressure, may lead to locational differences in the mechanical properties (Bower et al., 2024). Additionally, to enhance wearing comfort by ensuring close contact between the inner liner and scalp, different locations of the helmet shell were designed with varying geometric radii (Bland, McNally & Rowson, 2018; Spyrou, Pearsall & Hoshizaki, 2000). These shell-related factors may affect the transmission and attenuation of the impact forces. When force is transmitted to the liner, the cushioning foam within the liner absorbs a substantial portion of the impact energy through deformation and fracture, thereby dispersing the force across a broader area of the skull (Fernandes & De Sousa, 2013). Considering the design of ice-hockey helmets, different liner materials can be applied to various locations. For instance, the RE-AKT 95 helmet uses XRD foam at the side and top locations. The open-cell structure of the XRD foam allows air to flow between cells, causing the material to deform quickly when a load is applied, theoretically offering good energy absorption (Emerson et al., 2024). However, the experimental findings demonstrate that XRD foam attenuates linear acceleration less effectively than vinyl nitrile foam (Emerson et al., 2024). Additionally, differences in the liner ventilation design across helmet locations may affect heat dissipation and the available area for energy absorption within the liner (Bland, McNally & Rowson, 2018; Emerson et al., 2024; Forero Rueda & Gilchrist, 2012). Furthermore, although helmets of different sizes were properly fitted, different headforms may exhibit different responses to the same impact (Oeur, Gilchrist & Hoshizaki, 2019). In addition, the tightness of helmet fit was not quantified using mechanical measurements, and variations in liner compression across different locations of each helmet may lead to differences in the measured head responses (Jennings, Amini & Müftü, 2026). These liner-related factors may also affect the transmission and attenuation of the impact forces. After partial energy absorption by the liner, the remaining force was transmitted to the head, and the response of the injury risk metrics was affected by the anatomical structures of the skull and brain. Finite element analysis and primate studies have demonstrated that, without helmet protection, identical impact conditions applied to different head locations result in greater skull deformation, intracranial pressure, and brain shear stress at the side location, along with longer durations of unconsciousness (Tiernan & Byrne, 2019; Zhang, Yang & King, 2001). These biomechanical responses indicate that the side of the head is more vulnerable to injury when subjected to an impact. Under impactor velocities ranging from 5 to 7 m/s without helmet protection, the HIC ranking followed the order: side > top > front > back (Levadnyi et al., 2018). These findings are similar to our results, indicating that the top of the head presents a relatively high risk of injury, second only to the sides of the head. Compared to other locations, the geometry of the skull, local skull thickness, mechanical stiffness of the skull, and the distance from the impact point to the head center at the side and top may contribute to weaker mechanical resistance (Lianes et al., 2019; Liu et al., 2019; Thompson-Bagshaw, Quarrington & Jones, 2022; Zhang, Yang & King, 2001). This may decrease the ability of these locations to attenuate impact forces and help explain the HIC results observed in this study. In summary, the relatively higher HIC observed during impacts on the side and top locations of the helmet may be attributed to the interaction between locational variations in the helmet protective performance and structural differences between the skull and brain.

Helmets from three major manufacturers in the ice hockey equipment market were included in this study. Although the sample did not cover all helmet models, it represents a substantial portion of the current market, and the results therefore provide valuable reference information. The results indicate that impact energy increases with impact velocity, resulting in greater head responses during a single impact. A comparison of the consistency among HIC, SIR, and TSIR results shows that an increased seasonal head injury risk associated with weaker side protection is observed under low-velocity impact conditions. This finding suggests that helmet design should further optimize the energy-absorbing liner materials, structure, and thickness to better address low-velocity impacts (Haid et al., 2023). Furthermore, the consistency analysis indicates that impact frequency is the dominant contributor to seasonal head injury risk in most cases. Accordingly, ice hockey players may reduce injury risk by lowering the frequency of impacts from different directions through vision training, contact training, or practice contact restrictions (Antonoff et al., 2021; Pankow et al., 2022). Under medium- and high-velocity impact conditions, if high-frequency impacts can be more easily modified, reducing impacts to the back location should be prioritized. However, if altering impact frequency across locations is equally challenging, reducing impacts to the side of helmet may be more effective, as this location is associated with a higher injury risk during a single impact.

This study had several limitations. First, the current head model does not simulate the human neck, and the experimental results do not consider the influence of rotational motion on brain tissue injury. Linear acceleration has proven to be less predictive of head injuries than other biomechanical metrics, such as maximum principal strain, intracranial pressure, and brain shear stress. In addition, the compliance of the MEP used in the present study may differ from that of real-world impact objects encountered in sport, and the removal of the facemask during testing differs from real-world playing conditions, which may influence the resulting head response measurements. To enhance the accuracy of traumatic brain injury risk assessments, future studies may utilize more biofidelic anthropomorphic test devices together with more realistic impact materials used as the anvil to accurately record linear and rotational accelerations during head impacts. Furthermore, adopting models and simulation techniques that incorporate a wider range of personalized anatomical and biomechanical parameters could lead to more precise estimations of intracranial pressure and brain shear stress. Second, although statistically significant differences were observed between impact locations, the magnitude of these differences was relatively small and the values remained within a similar range. Therefore, these findings should be interpreted with caution, and further epidemiological studies are needed to confirm whether the observed statistical differences are clinically meaningful. Finally, in this study, ice hockey helmets from different brands and models were selected. Owing to stock limitations, helmet sizes were not standardized. Consequently, in addition to the primary variables (impact velocity, impact location, and environmental temperature), several potential confounding factors were introduced, including helmet manufacturer, helmet type, helmet size, and headform size. These factors may have negatively influenced the study outcomes. Future research should aim to minimize or control such confounding variables during experimental design to enhance the accuracy of the results.

## Conclusions

In conclusion, this study demonstrated that under low-velocity impacts at low and ambient temperatures, the significantly higher HIC observed in the side location compared to the back and front locations may be a key factor influencing the risk of head injuries. To enhance the prevention capability of ice hockey helmets, the optimization of the energy absorption capacity at the side location during a low-velocity impact is recommended.

## Supplemental Information

10.7717/peerj.21366/supp-1 Supplemental Information 1 TSIR, SIR, and HIC results calculated for the different ice hockey helmet models The head injury criterion (HIC) is calculated based on linear acceleration to predict the probability of AIS 2+ head injuries under a single impact. Seasonal injury risk (SIR) and total seasonal injury risk (TSIR) are injury probability metrics (%) derived from the HIC.

## Additional Information and Declarations

### Data Availability

The following information was supplied regarding data availability: The raw data is available in the Supplementary File. The raw data is available at Zenodo: – Rui, L. (2025). ice hockey impact data [Data set]. Zenodo. https://doi.org/10.5281/zenodo.17691341
